# Genetic Structure, Cytonuclear Discordance and Cryptic Biodiversity in the Rare Hawaiian Plants *Schiedea stellarioides* and *Schiedea spergulina*

**DOI:** 10.1002/ece3.74639

**Published:** 2026-11-13

**Authors:** Molly Nepokroeff, Bridget Jacobs, Lisa Wallace

**Affiliations:** ^1^ Department of Biology University of South Dakota Vermillion South Dakota USA; ^2^ Department of Integrative Biology University of Colorado‐Denver Denver Colorado USA; ^3^ Denver Botanic Gardens Denver Colorado USA; ^4^ South Heart School South Heart North Dakota USA; ^5^ Department of Biological Sciences Old Dominion University Norfolk Virginia USA

**Keywords:** chloroplast introgression, cryptic biodiversity, cytonuclear discordance, *Schiedea*

## Abstract

The endemic Hawaiian plant genus *Schiedea* (Caryophyllaceae) represents an adaptive radiation of 36 species. *Schiedea* species exhibit a wide range of morphologies, breeding systems, and habitats, making it an excellent model group for understanding insular evolution, including evolution of breeding systems in plants. Previous cytonuclear discordance in genealogies inferred for *Schiedea* species suggests multiple instances of past introgression. For example, two rare para‐ or sympatric species ( *S. spergulina* and *S. stellarioides* ) endemic to Kauai, which are placed in separate clades based on nuclear genealogies, were resolved as sister to each other in the reconstruction of chloroplast histories, suggesting chloroplast introgression. We examined the population structure of both species using nuclear and chloroplast microsatellites for three extant populations of *S. spergulina* and two extant populations of *S. stellarioides* , representing the remaining populations known of these species. Genetic variation in both species was low, and inbreeding coefficients are consistent with past population bottlenecks in both species. The nuclear microsatellite dataset indicated strong interspecific structuring (*F*_ST_ = 0.57) that is consistent with their divergence in previous nuclear phylogenies, with *S. spergulina* exhibiting significant population divergence (*F*_ST_ = 0.21), primarily due to isolation by distance. In contrast, the chloroplast microsatellite dataset indicated that the *S. spergulina* Waimea Canyon populations appear more similar to populations of *S. stellarioides* than to the conspecific Lawai Valley population. Approximate Bayesian Computation with Random Trees strongly supports a model of introgression from *S. stellarioides* into *S. spergulina* over incomplete lineage sorting for this pattern. These results indicate that the Lawai Valley population of *S. spergulina* is likely on a distinct evolutionary track relative to conspecific populations and may represent an ancestral lineage for this species. Given the divergence found in this study, continued protection of all populations for both species is important to preserve their genetic ancestry.

## Graphical abstract

The endemic Hawaiian plant species *Schiedea stellarioides* and *S. spergulina* have historically occurred partly in sympatry or parapatry on the island of Kawai, in the region of the Waimea Canyon. Populations of *S. spergulina* occurring in the Waimea Canyon exhibit introgressed chloroplast genomes from *S. stellarioides* occurring nearby, but does not exhibit nuclear introgression. However, a population of *S. spergulina* that is geographically remote from this location retains a unique and likely ancestral chloroplast genome for its species and is morphologically and genetically distinct, representing a cryptic lineage worth consideration of separate taxonomic status and additional conservation measures.

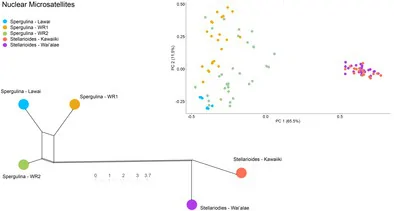

## 1 Introduction

Studies of the biota of the Hawaiian Islands have figured prominently in our understanding of adaptive radiations, speciation, and microevolutionary processes leading to allopatric divergence of populations (Baldwin and Wagner 2010; Dunbar‐Co and Wieczorek 2011; Garb and Gillespie 2009; Givnish et al. 2009; Jordan et al. 2005; Keeley and Funk 2011; McGlaughlin and Friar 2011; Nepokroeff et al. 2003; Price and Wagner 2018; Roderick and Gillespie 1998). While much attention has focused on deeper evolutionary processes and phylogenetic relationships of Hawaiian adaptive radiations, less attention has been given to more recent patterns of gene flow and allopatric divergence of populations of Hawaiian organisms (Dunbar‐Co and Wieczorek 2011; Pejhanmehr et al. 2024; Roderick and Gillespie 1998; Seeley et al. 2023; Wallace et al. 2009). Population genetic studies of Hawaiian organisms have demonstrated a number of common patterns, including isolation by distance (Dunbar‐Co and Wieczorek 2011; McGlaughlin and Friar 2011; Pejhanmehr et al. 2024), patterns that support the progression rule (Arnold et al. 2025; Wallace et al. 2009), and population bottlenecks due to population crashes for rare island species (Culley et al. 2025), among other patterns. Studies of population genetics of Hawaiian plants that integrate deeper‐level phylogenetics, show promise for identifying patterns of cryptic diversity which may be useful for conservation planning and assessment of biodiversity in this imperiled flora.

Hybridization occurs when gene flow occurs between divergent populations or species and can lead to introgression, the permanent incorporation of genes from one species or population into the gene pool of another through repeated backcrossing of a hybrid with one of its parent species, leading to retention of genetic variation from the donor. Hybridization and introgression are multifaceted processes that can facilitate adaptive evolution (Folk et al. 2018; Penalba et al. 2024). Traditionally viewed as detrimental to speciation, hybridization and introgression can either enhance or prevent evolutionary diversification, but their preventative powers (i.e., loss of fertility associated with hybrid sterility) have historically been emphasized (Bell and Travis 2007). In contrast, other work has focused on the role of interspecific gene flow in facilitating diversification in adaptive radiations (Marques et al. 2019; Seehausen 2004). In particular, the role of interspecific gene flow and transgressive segregation in creating unique and diverse characteristics that allow species to radiate and colonize diverse ecological niches in adaptive radiations has been suggested (Seehausen 2004). Interspecific gene flow has been implicated in the majority of classic examples of animal and plant adaptive radiations including the Lake Tanganyika Cichlid tribe (Day et al. 2007), Darwin's finches in the Galapagos Islands (Grant et al. 2004) and the Hawaiian silverswords (Barrier et al. 1999; Baldwin et al. 2021), among others.

The introgression of cytoplasmic genomes (i.e., plastid and mitochondrial) is often asymmetric and very rapid (Currat et al. 2008). Although chloroplast introgression has previously been attributed to positive selection of locally adapted plastid genomes, simulations by Currat et al. (2008) suggest that plastid introgression can occur through demographic imbalance during the initial advance of an invading species into the range of resident species. Chloroplast introgression has often been suggested when there is cytonuclear discordance or when phylogenies inferred from chloroplast vs. nuclear partitions reflect different patterns. Cytonuclear discordance is well reported for many plant groups (Folk et al. 2018; Lee‐Yaw et al. 2019), but it is important to note that disparate phylogenetic histories can also result from other processes such as incomplete lineage sorting and convergent selection. Cytonuclear discordance due to plastid introgression is prominent in several groups of Hawaiian plants including silverswords (Baldwin et al. 2021), *Melicope* (Paetzold et al. 2019), *Plantago* (Dunbar‐Co et al. 2008), *Euphorbia* sect. *Chamaecyse* (Yang et al. 2018), and *Schiedea* (Willyard et al. 2011), among others. Some authors suggest that plastid genomes can introgress and become fixed under conditions common on islands, where a colonist can invade the range of a closely related species. Such introgression has been associated with habitat shifts, as in silverswords (Baldwin et al. 2021), with the potential for selection for the invading species to drive the home species plastid genome to fixation in a novel habitat where there may be a selective advantage for that plastid haplotype. Lee‐Yaw et al. (2019) found evidence that cytoplasmic introgression and positive selection contributed to cytonuclear discordance in annual sunflowers, demonstrating the complexity of evolutionary processes that have contributed to contemporary plant populations.

The genus *Schiedea* (Caryophyllaceae) represents an adaptive radiation of 36 extant species (Wagner et al. 2024) that are endemic to the Hawaiian Islands and have been widely studied as a model group for investigating the evolution of plant growth form, habitat preference, plant breeding systems (Wagner et al. 2005; Sakai et al. 2006; Weller et al. 1998), floral development (Harris et al. 2012), floral volatiles (Powers et al. 2022), pollination biology (Weller et al. 2017), and conservation (Sakai et al. 2018). *Schiedea* is one of the five largest radiations of Hawaiian plant species resulting from a single colonization event (Price and Wagner 2011) after the endemic Lobelioideae (125 species), *Cyrtandra* (Gesneriaceae, 58 species), *Melicope*/*Platydesma* (Rutaceae, 52 species), and is similar in size to the Hawaiian silverswords (Asteraceae, 33 species). *Schiedea* includes both deciduous and non‐deciduous perennials, sprawling vines, and woody subshrubs, and occupies a range of habitats, including coastal shorelines, lowland and montane mesic forests, wet rainforests, and subalpine habitats on all the main Hawaiian Islands. *Schiedea* exhibits the greatest diversity of breeding systems of any native Hawaiian plant genus, including both outcrossing and selfing hermaphrodites, mixed mating systems, gynodioecy, subdioecy, and full dioecy (Wagner et al. 2005). This range of breeding systems and their correlation with specific habitat types suggests that breeding systems may have evolved in response to habitat shifts during the diversification of the genus in Hawaii (Weller et al. 1998).

Phylogenetic relationships within *Schiedea* have been studied both on a molecular and morphological basis, and have clarified the major lineages within *Schiedea*, subgeneric and sectional groupings, and direction of colonization within the Hawaiian Islands (Wagner et al. 2005; Willyard et al. 2011). Wagner et al. (2005) recognized eight taxonomic sections based on phylogenetic relationships and morphology. Many of the recognized sections reflect similar morphology, breeding systems, pollination mode, and ecological adaptations. Analyses of nuclear and chloroplast genealogies have begun to clarify the relationships within this recently diverged genus, but they have also brought to light cryptic biodiversity and reticulate events as important factors in the evolutionary history of the *Schiedea* radiation. Interspecific hybridization based on morphological intermediates is documented between five *Schiedea* species pairs (Wallace et al. 2011; Weller et al. 2001; Willyard et al. 2011), but artificial hybrids were successfully created in pairwise crosses of 17 species (Weller et al. 2001), and some species of *Schiedea* are known to form long standing hybrid zones (Powers et al. 2022). Willyard et al. (2011) identified at least four instances of cytonuclear discordance, and of those, two cases (that of *S. lydgatei* × *S. sarmentosa* and *S. hookeri* × *S. mannii* ) also show evidence of morphological intermediacy in the field, while two others (that of *S. globosa* × *S. adamantis* and *S. spergulina* × *S. stellarioides* ) do not exhibit morphological intermediacy in the field. One of the most well‐supported cases in the work of Willyard et al. (2011) that is of particular interest is *Schiedea stellarioides* H. Mann and *Schiedea spergulina* A. Gray, which are both endemic to the island of Kauai (Wagner et al. 2005). Several *S. spergulina* accessions grouped with *S. stellarioides* (Willyard et al. 2011), an unusual pattern given that these species differ markedly morphologically, have been placed taxonomically in section *Mononeura* and section *Schiedea*, respectively (Wagner et al. 2005), and are well‐supported as members of different clades in nuclear phylogenies (Willyard et al. 2011). Because plastid loci are known to be introgressed more readily than nuclear loci (Currat et al. 2008) and are known to be introgressed among sympatrically distributed taxa, we hypothesized that the chloroplast genome of *S. stellarioides* may have been introgressed into *S. spergulina* on Kauai.

*Schiedea stellarioides* is federally listed as endangered (Mehrhoff et al. 1994). This species is a hermaphroditic subshrub characterized by pendant inflorescences and distinctive, four‐ridged stems as well as coral‐colored anthers. Once thought to be extinct, a population of *S. stellarioides* was rediscovered in 1991 growing in montane mesic forest on the island of Kauai and is now known from two populations that occur in fairly close proximity near Waialae Falls on central Kauai (Lorence et al. 1995). *Schiedea stellarioides* was historically distributed on the western side of Kauai, from Waimea, O'paewela, Koholamanu, and also on cliffs above Hanakapai Beach on the Napali coast, and a single collection from the southeastern side of the island at Haupu Ridge. The locations of historical (extant and extirpated) populations of *S. stellarioides* are shown in Figure 1.

**FIGURE 1:**
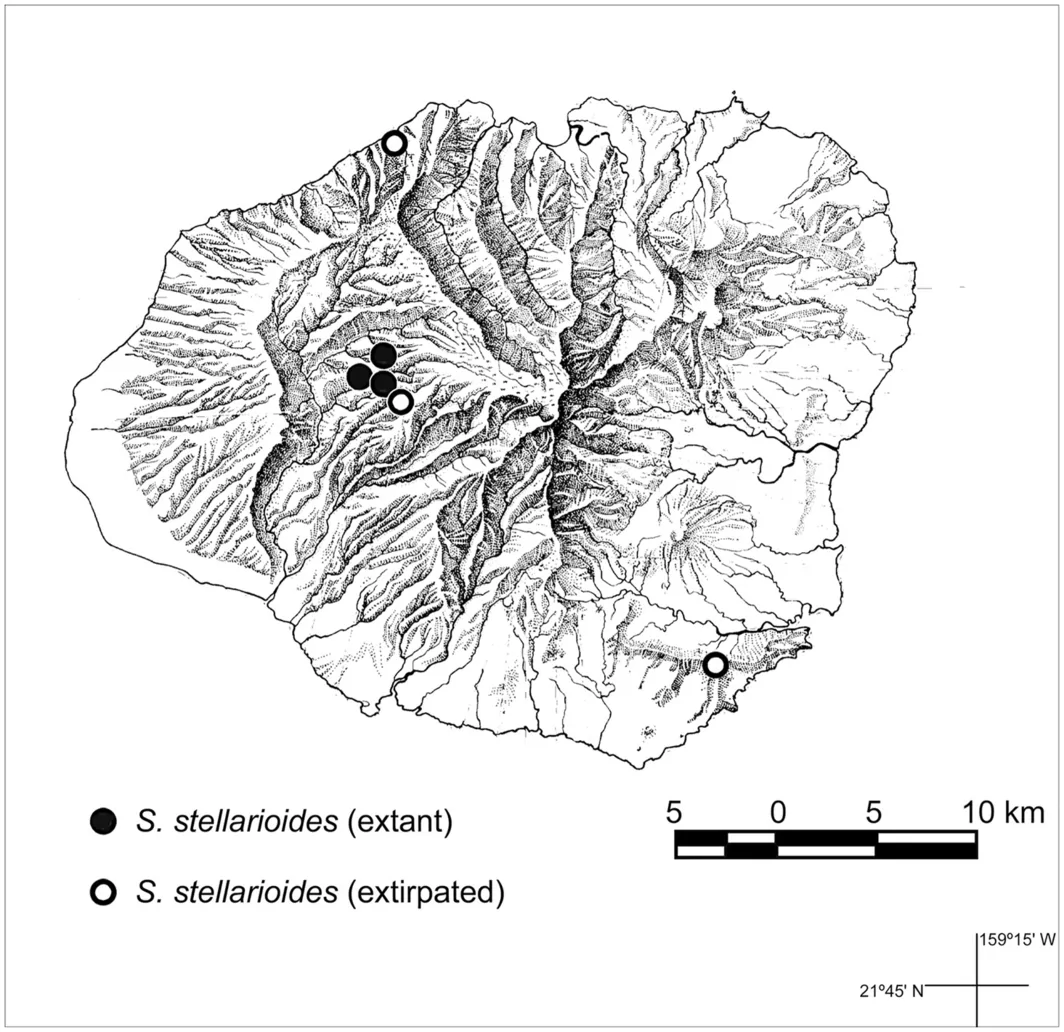
Historical (extant and extirpated) populations of *S. stellarioides* as published in Wagner et al. (2005), used with permission of the authors and Systematic Botany Monographs. Field work for our study now indicates only two extant populations shown in Figure 3.

*Schiedea spergulina* is a dioecious sprawling shrub that is endemic to dry, windswept cliffs and lowland sites on Kauai. Formerly more widespread in western Kauai on dry cliff faces and immediately adjacent cliff tops, *S. spergulina* is now only found in isolated populations on the sheer vertical walls of Waimea Canyon in central Kauai, with another extant disjunct population located approximately 30 miles south and east of Waimea Canyon on the cliffs of the Lawai Valley in southern Kauai. *Schiedea spergulina* formerly occurred around the localities of Olokele Canyon, Hanapepe, Wahiawa, Waialae Canyon and Kalalau and Hipalau Valleys on Kauai, but is now extirpated. Figure 2 shows the extant and extirpated populations of *S. spergulina* species as published in Wagner et al. (2005), however, field studies conducted in 2008 revealed only three extant populations of *S. spergulina* (Figure 3). As a result of its rarity, *S. spergulina var. leiopoda* is federally listed as endangered and *S. spergulina var. spergulina* is listed as threatened (Mehrhoff et al. 1994; USFWS 1994) [Note: While previous taxonomic treatments of *S. spergulina* recognized infraspecific taxa (Sherff 1945; Wagner et al. 1999), a single species concept was adopted by Wagner et al. (2005) who cited overlapping variation in traits of the varieties].

**FIGURE 2:**
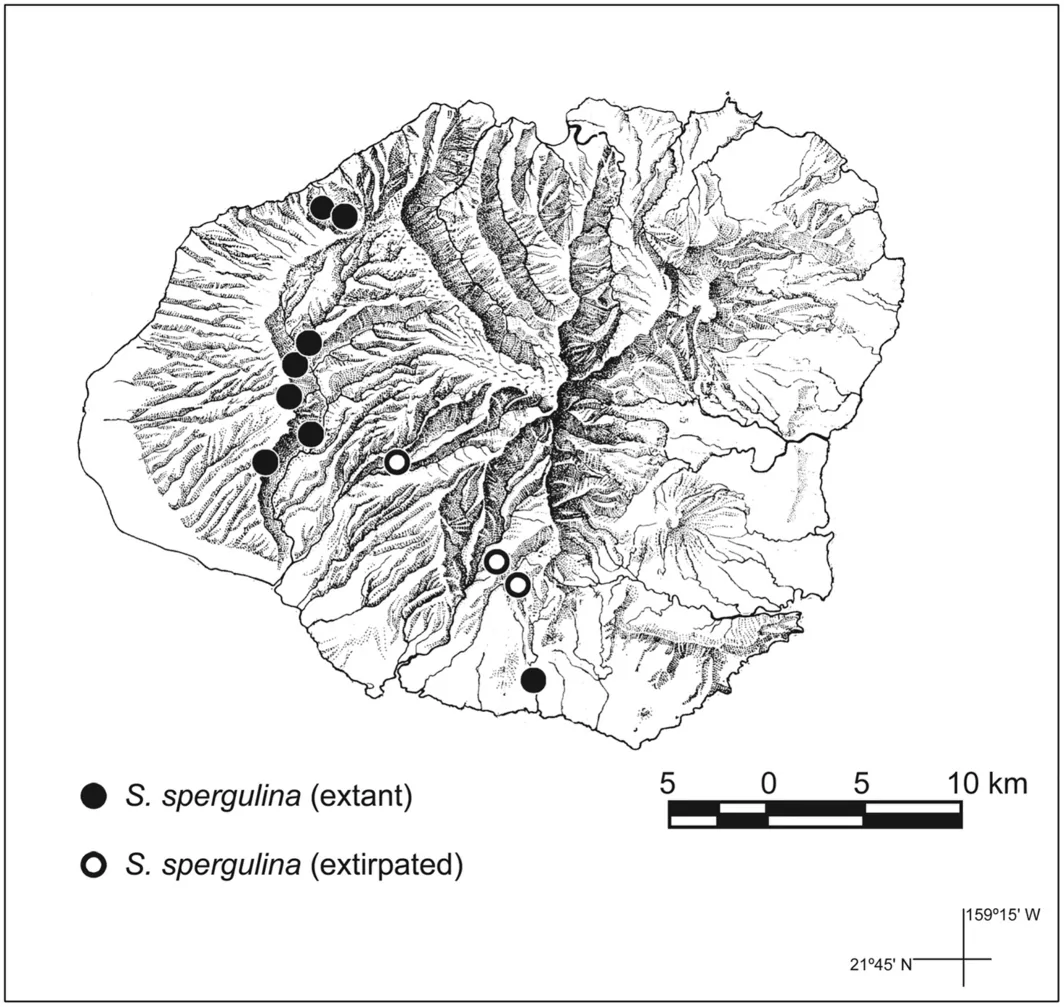
Historical (extant and extirpated) populations of *S. spergulina* (adapted from Wagner et al. (2005), used with permission of the authors and Systematic Botany Monographss).

**FIGURE 3:**
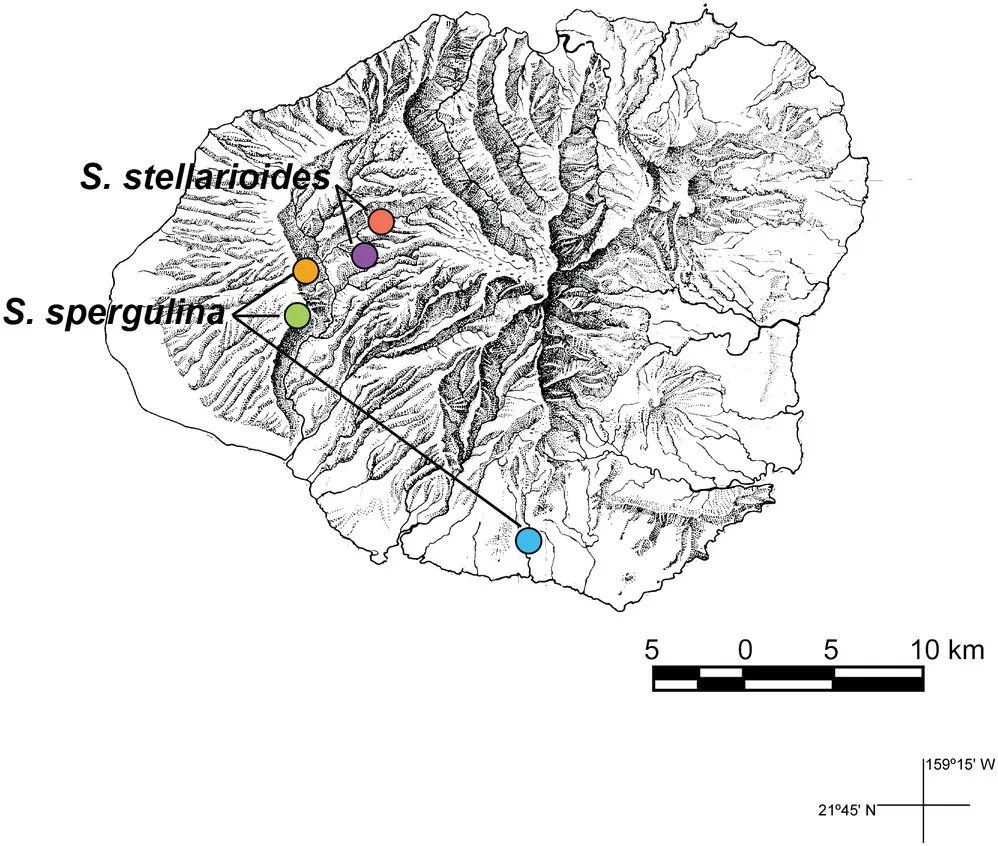
Location of extant populations of *S. stellarioides* and *S. spergulina* on the island of Kaua'i. Colors correspond to NeighborNet and PCA analyses in Figure 5A,B.

This study describes a deeper investigation of the cytonuclear discordance revealed by Willyard et al. (2011) for *S. spergulina* by addressing (1) whether a shared nuclear history is more readily detectable at the population level for *S. stellarioides* and *S. spergulina* on Kauai with microsatellite loci, (2) the extent of shared plastid variation in populations of *S. spergulina* , and (3) if cytonuclear discordance is more likely to be the result of introgression or incomplete lineage sorting. Additionally, we discuss population genetic structure, connectivity, and diversity in these federally listed rare species and revisit varietal delimitations in *S. spergulina* .

## 2 Materials and Methods

### 2.1 Population Sampling

Fresh leaf tissue was collected from all known extant populations of *S. spergulina* and *S. stellarioides* . Three populations of *S. spergulina* were sampled, two in Waimea Canyon, designated as Waimea Rim 1 (WR1; located at ca. mile marker 5.7 on state highway 550 on Kauai) and Waimea Rim 2 (WR2; located at 21°59′28.638″N, 159°40′18.684″W); and one from the Lawai Valley population (Lawai) located on the property of National Tropical Botanical Garden, from a cliff above Medicinal Garden (a population formerly recognized as *S. spergulina var. leiopoda* ). *Schiedea stellarioides* was sampled from the only two known extant populations of that species, both near Wai'alae Falls in the interior highlands of Kauai and are designated as Kawaiki (located at Ridge south of Kawaiiki Valley, 22°05′3.5″N, −159°36′16.06″W) and Waialae (located along north slopes of Waialae Ridge, 22°04′26.68064″N, −159°36′7.14941W). Collection localities for both species are shown in Figure 3. Sampled populations ranged in size from 19 to 33 individuals. Leaves were stored in silica gel until processed.

### 2.2 DNA Extraction and Nuclear and Chloroplast Microsatellite Genotyping

Genomic DNA was extracted from leaf tissues using the DNeasy Plant Kit (Qiagen) according to the manufacturer's protocol. PCR amplification of nuclear microsatellite loci used primers developed for *Schiedea* (Culley et al. 2008) and are shown in Table 1. Amplification was done using a Multiplex PCR Kit (Qiagen) with two reactions amplifying six and four loci, respectively. Fluorescent labels 6‐FAM, NED, VIC, and PET were added to each reaction, with each locus distinguished by label and/or non‐overlapping size. PCR amplifications were run with a 10 μL reaction volume as follows: 5 μL Qiagen master mix, 1.25 μL 10 mM primer mix, 3 μL of ddH20, and 1 μL of DNA. The thermal cycler program was 95°C for 15 min, then 94°C for 30 s, 57°C for 90 s, and 72°C for 60 s repeating for 30 cycles, then 8 cycles of 94°C for 30 s, 53°C for 90 s, 72°C for 60 s, and finally a 10 min extension at 72°. After PCR, amplification was checked on a 1.5% agarose gel stained with SYBR Green (Invitrogen).

**TABLE 1:** Microsatellite loci, primers, and label used for 8 nuclear microsatellites (Culley et al. 2008) and five chloroplast microsatellites (Kuenzi 2010).

Locus name	Primer sequence (5′–3′)	Size (bp)	Label	Number of alleles
Nuclear microsatellite loci				
SA05	F: TGTCAACCTCATGACATGGT	184–217	VIC	11
	R: AGAGCAGCAACAGGGAGAAG			
SA06	F: CCCACCTGCATGAATACTGA	188–203	NED	3
	R: TACTGCCCAACTGCCCTATC			
SA15	F: CCCACTAAATCACTAGCACATTTC	171–195	NED	8
	R: ACCATCACTGCCTCAGATCC			
SA30	F: TAAGTGGGAAGTGGGAAAATG	264–270	VIC	4
	R: GTGGTGAGATTCAACAAGGTG			
SA31^a^	F: GGCGATTATTGACTCATCTCCA	208–241	VIC	6
	R: CCACTCTCACCTCACCCAAC			
SA32	F: CGCCTTCGACTCGTCTTAGT	188–206	6‐FAM	4
	R: CACTGCTACGACTCGGCTTA			
SA36	F: CATCTTTAATTGACGCCTTGG	249	6‐FAM	1
	R: AACCGAAATGTCGAATCATTG			
SA38	F: TCAAACCAAGTCCAGTATTCG	188–197	PET	3
	R: TGAGAACTCGGGTTATGCTG			
Chloroplast microsatellite loci				
Schcp14	F: TGGCTTGAGCCTTAGTATGGA	201–204	6‐FAM	4
	R: ACAACACAGCACAGCCAATC			
Schcp17	F: CCCATTCTCGGTTTATAGCC	174–180	PET	2
	R: GACACAGCAAGGCAGTTTCA			
Schcp18	F: AGCCTGAAATCGATCCAATC	154–161	NED	2
	R: TTCTGACACAGCAAGGCAGT			
Schcp21	F: TGACTTTTTGTTTTAGGGGTTTG	134–144	VIC	3
	R: CCCTGTAATCAGGACTCCTTTG			
Schcp22	F: TAGCGGCTCGGTTCTAAGTG	96–98	6‐FAM	3
	R: AATTCAAAAGATCCGTTCAGG			

*Note:* Numbers reported in the ‘size’ and ‘number of alleles’ columns reflect the data from the current study.

^a^ SA31 was only amplified in *S. spergulina* and was not able to be amplified in *S. stellarioides* .

Successful amplification products were visualized using an Avant 3100 Genetic Analyzer (Applied Biosystems), and fragment sizes were determined by comparison to the LIZ500 molecular size marker (Applied Biosystems) in Gene Mapper software (Applied Biosystems). Seven loci were successfully amplified for all populations of both species, and one additional locus was amplified for all populations of *S. spergulina* .

Variation in the chloroplast genome was evaluated by genotyping samples at five chloroplast microsatellite regions. Amplification primers were developed by Kuenzi (2010) for *Schiedea*, and these markers span the chloroplast regions *trnL, psbE/petL* (2 loci), *psbM/trnD*, and *trnT/trnD*. Four loci (Schcp14, 17, 18, 22) were amplified in a multiplex reaction, while Schcp21 was amplified singly. One primer of each set was labeled with a fluorescent tag (6‐FAM, PET, NED or VIC; see Kuenzi 2010 for details). Each sample was amplified in 10 μL volume containing 1X buffer (New England Biolabs), 0.16 mM dNTPs, 2.5 mM MgCl_2_, 2 μM of each primer, 0.5 unit of Taq polymerase (New England Biolabs), and 0.5 μL DNA template. The thermal cycler program of the 4‐locus multiplex was 95°C for 3 min, 35 cycles of 95°C for 15 s, 60°C for 30 s, 72°C for 30 s, and a final extension step at 72°C for 1 min. The program for Schcp21 was 95°C for 15 min., 35 cycles of 94°C for 30 s, 57°C for 45 s, 72°C for 45 s, followed by 1 cycle of 94°C for 30 s, 53°C for 45 s, 72°C for 45 s, and a final extension at 72°C for 10 min. Successful amplification products were then visualized using an Applied Biosystems 3730 system, and fragment sizes were determined by comparison to the LIZ600 molecular size marker (Applied Biosystems) using GeneMarker (SoftGenetics). All chloroplast loci were successfully amplified in both species.

### 2.3 Statistical Analyses

#### 2.3.1 Genetic Variation and Population Structure at Nuclear Microsatellite Loci

For each population the number of observed alleles was calculated along with the number of private alleles at each locus using GenAlEx v. 6.4 (Peakall and Smouse 2006). Two additional people re‐scored all private alleles to confirm that they were indeed private alleles and not scoring errors. Hardy–Weinberg Equilibrium was checked by calculating and comparing the expected (H_E_) and observed (H_o_) heterozygosity values, and the inbreeding coefficient (*F*_IS_) and a fixation index (*F*_ST_) were calculated using FSTAT v.2.9.3.2 (Goudet 1995). Using GenAlEx, a pairwise genetic distance matrix was generated for individuals and used in a Principal Coordinates Analysis to evaluate the relatedness of genotypes between populations. SplitsTree (Huson and Bryant 2024) was used to generate a network of relatedness using the NeighborNet function with a matrix of mean individual pairwise codominant genotypic distances for populations calculated using GenAlEx 6.5 (Peakall and Smouse 2006).

Individual population membership and population structure were analyzed using the Bayesian analysis of STRUCTURE vers. 2.0 (Pritchard et al. 2000). Due to the close proximity of several populations both within and between species, an admixture model was used. Each run had a burn‐in of 25,000 and a run length of 75,000 after burn‐in. Methods described in Evanno et al. (2005) were used to determine the “true” number of populations (K). Analyses of molecular variance (AMOVA; Excoffier et al. 1992) were used to determine if the species maintain significantly different allele frequencies and thus exhibit population genetic structure. Three analyses (between the species and within each species) were conducted using pairwise genetic distances and 50,000 permutations of the data to test for significance. All analyses were conducted in Arlequin v. 3.1 (Excoffier et al. 2005).

Recent bottlenecks in all of the populations were evaluated using BOTTLENECK (Cornuet and Luikart 1996; Piry et al. 1999). In this analysis, the two‐phase model (di Rienzo et al. 1994) was used with 95% single‐step mutations and a variance of 12 among multiple steps, following the recommendations of the authors (Piry et al. 1999). Loci that exhibited significant deviations from Hardy–Weinberg equilibrium were not considered in these analyses. Wilcoxon's sign rank test was used to detect significant excesses of heterozygosity as evidence of a genetic bottleneck across all loci within each population.

BAYESASS v. 1.3 (Wilson and Rannala 2003) was used to estimate migration rates between all pairs of populations. The analysis was run for 20 million generations with sampling every 2000th generation. The first 2 million cycles were discarded as burn‐in. Five duplicate analyses with different random seeds were run to ensure convergence in the estimated migration rates. Results are reported from a single run, although all runs produced nearly identical values. To evaluate robustness of the point estimates, estimated migration rates were compared to a null model of no information on migration. Thus, significant migration rates were inferred only when the estimated values resided outside of the confidence intervals of point estimates in the absence of informative data.

#### 2.3.2 Genetic Variation and Population Structure at Chloroplast Microsatellite Loci

Using fragment sizes of the amplified chloroplast microsatellite loci, the number of haplotypes, number of private haplotypes, and haplotypic diversity in each population and species were calculated with HaplotypeAnalaysis v1.05 (Eliades and Eliades 2009). Principal Coordinates Analysis based on pairwise genetic distances of individuals was used to evaluate the relatedness of individuals based on plastid genotypes. SplitsTree (Huson and Bryant 2024) was used to generate a network of relatedness among the unique haplotypes using the NeighborNet function and a matrix of pairwise p‐distance.

#### 2.3.3 Coalescent Simulations to Evaluate Introgression and Incomplete Lineage Sorting

Approximate Bayesian computation via Random Forests (ABC‐RF) was used to evaluate alternative scenarios of incomplete lineage sorting (ILS) and unidirectional introgression as the cause of cytonuclear discordance between the two microsatellite datasets. diyABC‐RF (Collin et al. 2021) was used to simulate 60,000 total datasets with similar properties to our observed datasets based on three scenarios: (1) incomplete lineage sorting that dates to the ancestral population of both species, (2) introgression from *S. stellarioides* into the *S. spergulina* WR1 and WR2 populations as separate events, and (3) introgression into an ancestral *S. spergulina* WR population prior to it splitting into separate populations recognized as WR1 and WR2 today. We allowed the size of each population to be independent and modeled a single ancestral population of *S. stellarioides* , given the absence of strong genetic divergence observed in our datasets, before allowing gene flow from this species into *S. spergulina* . In scenario 2, the rates of introgression into WR1 and WR2 were independent, and we set the timing of coalescence of the two populations of *S. stellarioides* and coalescence of the two WR populations to be similar. Priors for the simulations were set as follows: N: 10–10,000; t: 10–1 million generations considering a generation time of 5 years; rates of introgression from 0.01 to 0.99. Random forests were generated with 500 trees and used to compare summary statistics of the simulated and observed datasets in selecting the best‐fitting model.

## 3 Results

### 3.1 Nuclear Microsatellite Allelic Variation

Six of the seven sampled nuclear microsatellite loci were found to be polymorphic. In total, 34 alleles were identified in the 7 loci across all 136 individuals (Table 2). Locus SA36 was fixed for a single allele in all individuals. One additional polymorphic locus (SA31) was able to be amplified in *S. spergulina* , yielding 7 additional alleles for a total of 41. However, this locus did not amplify in *S. stellarioides* , thus locus SA31 was not included in combined analyses of the two species. A total of 29 alleles were observed among 89 individuals, for the three populations of *S. spergulina* , and 26 alleles were found for 44 individuals of the two populations of *S. stellarioides* (Table 2). The total number of alleles per locus for both species ranged from the 1 at SA36 to a high of 11 alleles at SA05, and an average of 4.86 alleles per locus. The number of alleles per population had a high and low in *S. spergulina* with the Lawai population exhibiting 15 total alleles and the WR2 population exhibiting 24 total alleles.

**TABLE 2:** Summary of allelic diversity, polymorphism, heterozygosity, and fixation indices for nuclear and chloroplast microsatellites in two populations of *S. stellarioides* and three populations of *S. spergulina* .

Population	Wai'alae	Kawaiiki	*S. stellarioides*	Lawai	WR1	WR2	*S. spergulina*
Nuclear microsatellites							
N	25	19	44	26	30	33	89
A	23	20	26	15	21	24	29
PA	6	3	12	2	2	5	8
POLY	5	5	6	3	5	6	7
% POLY	71.43	71.43	85.71	37.50	62.50	75.00	87.50
H_O_	0.234	0.241	0.341	0.183	0.271	0.303	0.257
H_E_	0.327	0.336	0.430	0.181	0.306	0.367	0.335
*F*_IS_	0.287	0.289	0.288	−0.100	0.115	0.177	0.122
*F*_ST_			0.021				0.142
Chloroplast microsatellites							
N	28	19	47	26	31	34	91
A	6	3	6	2	3	2	5
PA	3	0	6	2	1	0	5
H	0.693	0.649	0.711	0.077	0.239	0.258	0.544

*Note:* Values are based on seven nuclear loci amplified in *S. stellarioides* , eight nuclear loci in *S. spergulina* , and five chloroplast loci that amplified in both species.

Abbreviations: % POLY, percentage of polymorphic loci; A, number of alleles; *F*_IS_, inbreeding coefficient; *F*_ST_, population differentiation; H, haplotype diversity; H_E_, expected heterozygosity; Ho, observed heterozygosity; *N*, sample size; PA, number of private alleles; POLY, number of polymorphic loci.

### 3.2 Nuclear Microsatellite Genetic Structure and Population Membership

Strong genetic structuring was observed between the species, as indicated by AMOVA, STRUCTURE, PCoA, and network analysis. AMOVA indicated a greater amount of the observed variation in the nuclear microsatellite loci (57%) was found between the species than within them (Table 3). Within each of the species, variation in the nuclear loci primarily resides within populations (79%–98%), although *S. spergulina* had a significant amount among populations (21%) as well.

**TABLE 3:** Partitioning of genetic variation between *S. spergulina* and *S. stellarioides* and within each species based on analyses of molecular variance (AMOVA) using pairwise genetic distances between individuals with nuclear microsatellites.

Source of variation	DF	Variance	Percent of variation
*S. spergulina* – *S. stellarioides*			
Between species	1	1.36083	57.01*
Within species	264	1.02624	42.99
*S. spergulina*			
Among populations	2	0.22067	21.42*
Within populations	175	0.80933	78.58
*S. stellarioides*			
Among populations	1	0.02137	1.81
Within populations	86	1.15737	98.19

*Note:* An asterisk after percent of variation indicates significant structure (*p* < 0.05) at that level; significance values are based on 50,000 permutations of the data.

Abbreviation: DF, degrees of freedom.

Bayesian analyses performed with STRUCTURE on the nuclear microsatellite data indicated an optimal solution of *K* = 2 for the combined nuclear microsatellite data set based on the largest ΔK value. The assignment of individuals into two discrete clusters (Figure 4A) is consistent with species' boundaries. Additional analyses of *S. spergulina* and *S. stellarioides* recovered three distinct clusters in the former (Figure 4B), but in the latter (Figure 4C). Within *S. spergulina* , the three clusters correspond to the three populations, but this analysis suggests the possibility of inter‐population gene flow between WR1 and WR2.

**FIGURE 4:**
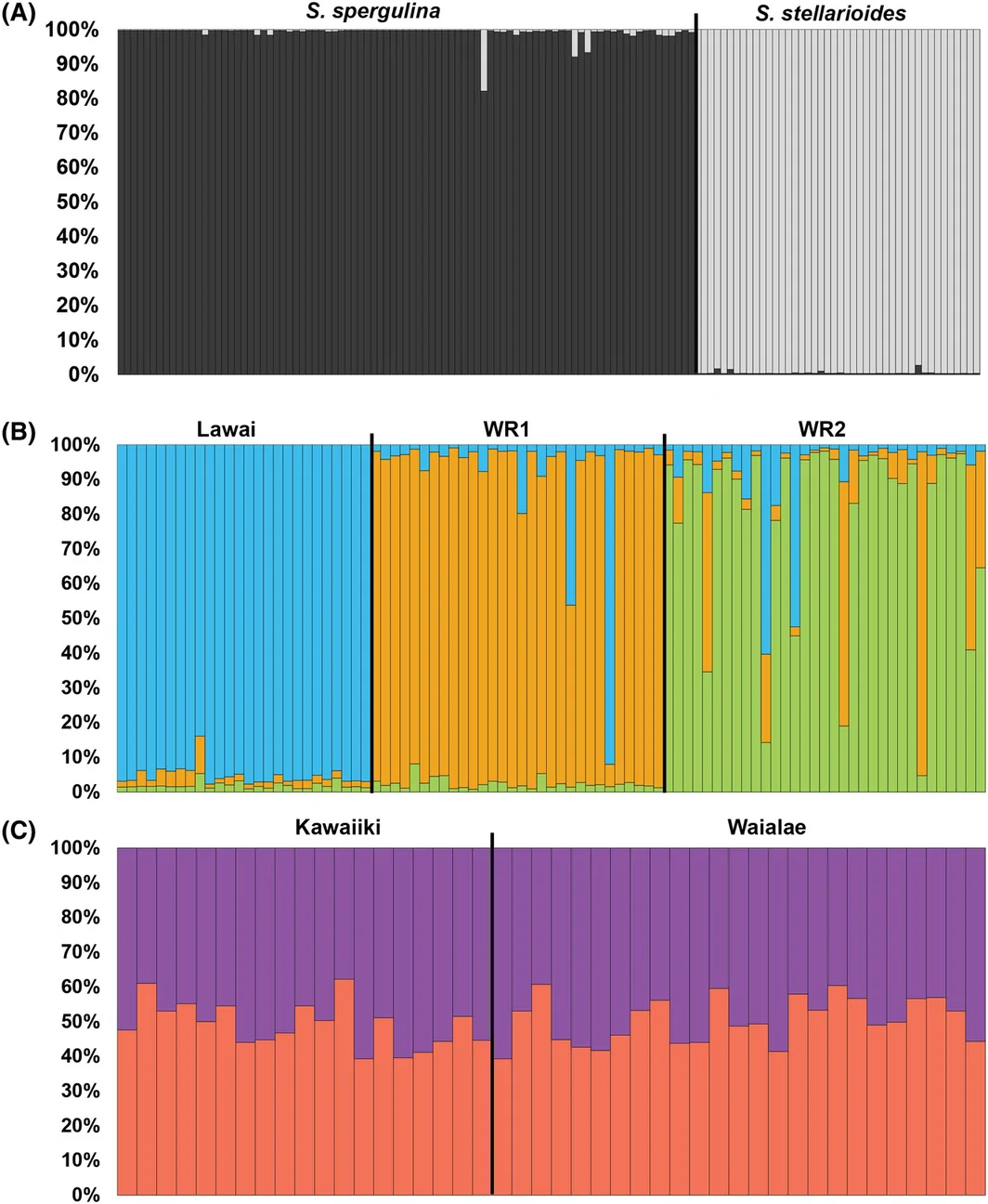
Results of STRUCTURE analyses for (A) *Schiedea spergulina* and *Schiedea stellarioides* . (B) Three populations of *Schiedea spergulina* . (C) Two populations of *Schiedea stellarioides*.

Principal Coordinates Analysis of all five populations based on nuclear microsatellite data revealed a clear distinction between *S. spergulina* and *S. stellarioides* , with the first and second axes explaining 76.96% of the total variation (Figure 5A). Within the species, the results were similar to the STRUCTURE analysis. For *S. spergulina* , there was a large degree of overlap between the WR1 and WR2 populations along Waimea Canyon Rim, but the Lawai population overlapped only with samples from WR2 (Figure 5A). Also mirroring the STRUCTURE analysis, samples of the two populations of *S. stellarioides* were not readily distinguishable in the PCoA plot (Figure 5A). The network of population relationships also supported a strong divergence between the two species (Figure 5A).

**FIGURE 5:**
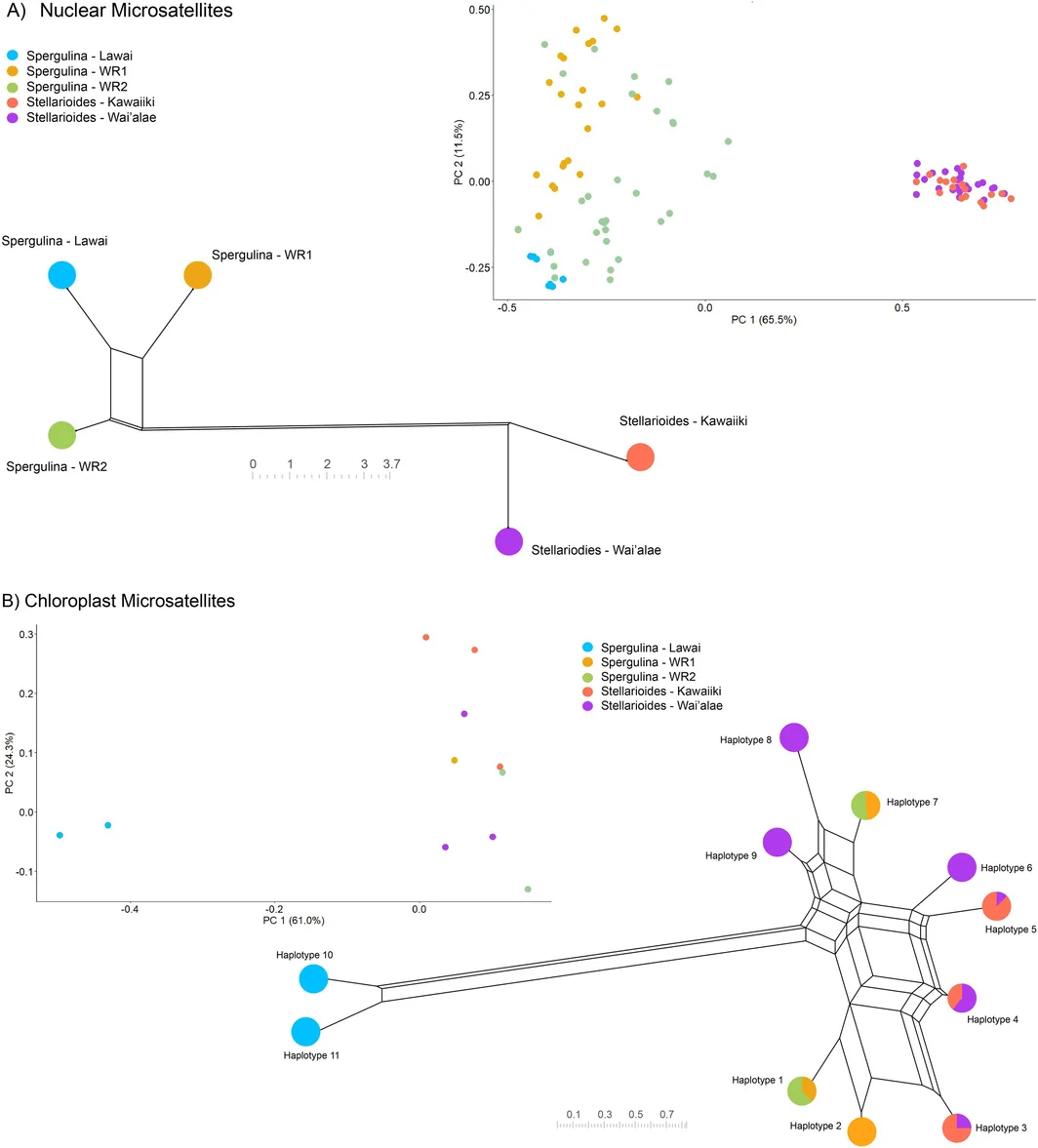
Relatedness of populations and individuals of *Schiedea spergulina* and *S. stellarioides* based on nuclear microsatellites (A) and chloroplast microsatellites (B). Networks were generated with the NeighborNet method and pairwise population genetic distances. Principal Coordinates Analysis plots depict patterns across the first axes. In B, only distinct chloroplast haplotypes are shown in the PCoA plot and network.

Evidence of recent bottlenecks based on variation in the nuclear microsatellite loci was found in all populations of both species and at multiple loci (Table 4). In the Lawai population of *S. spergulina* , all loci had an excess of heterozygous individuals, whereas in all other populations 3–4 loci exhibited heterozygote excess consistent with past bottlenecks. Contemporary gene flow was not suggested to be widespread according to the BAYESASS analysis (Table 5) of nuclear microsatellite variation. The only significant estimate of migration differing from zero was between the two populations of *S. stellarioides* , and this analysis suggested that the direction of gene flow was from the Wai'alae population into the Kawaiiki population.

**TABLE 4:** Results from tests of past bottlenecks for nuclear microsatellites performed with the software Bottleneck.

Population	No. loci with heterozygote excess	*p*‐value
*S. spergulina*		
Lawai	7	0.004
WR1	3	0.004
WR2	4	0.008
*S. stellarioides*		
Wai'alae	3	0.019
Kawaiiki	3	0.039

*Note:* Reported are the number of loci exhibiting significant heterozygosity excess and the *p*‐value for the Wilcoxon sign rank test, which is a global test over all loci.

**TABLE 5:** Estimated migration rates between all pairs of populations based on an analysis in BAYESASS for nuclear microsatellite data.

	Into					
	*S. stellarioides*			*S. spergulina*		
	Population	Wai'alae	Kawaiiki	Lawai	WR1	WR2
From	Wai'alae	0.931 (0.817–0.999)	**0.203 (0.002–0.322)**	0.003 (0.0000005–0.018)	0.003 (0.0000006–0.016)	0.003 (0.0000007–0.018)
	Kawaiiki	0.057 (0.0000006–0.168)	0.773 (0.668–0.985)	0.003 (0.0000003–0.018)	0.003 (0.0000006–0.018)	0.003 (0.0000009–0.019)
	Lawai	0.004 (0.0000006–0.022)	0.008 (0.00001–0.043)	0.987 (0.956–0.999)	0.005 (0.0000005–0.028)	0.008 (0.0000009–0.045)
	WR1	0.004 (0.0000003–0.021)	0.008 (0.00002–0.039)	0.003 (0.0000005–0.019)	0.986 (0.950–0.999)	0.013 (0.000002–0.085)
	WR2	0.004 (0.0000006–0.020)	0.008 (0.00002–0.041)	0.003 (0.0000007–0.021)	0.004 (0.0000004–0.023)	0.972 (0.880–0.999)

*Note:* For comparison, the estimate for migration with five populations in the absence of any informative data for five populations is 0.0417 (95% CI: 0.0000179–0.185). Values should be read as migration from the populations listed in the left column into populations in the top row. The single estimate of significant migration between two populations is shown in bold.

### 3.3 Chloroplast Microsatellite Variation

As in the nuclear dataset, chloroplast microsatellite variation was higher in *S. stellarioides* than in *S. spergulina* (Table 2). A similar number of haplotypes was observed between the species (i.e., 5 in *S. spergulina* and 6 in *S. stellarioides* ), but haplotype diversity was 0.544 and 0.711, respectively. Although the species did not have any haplotypes in common, those observed in the two Waimea Canyon (WR) populations of *S. spergulina* are more similar to haplotypes observed in *S. stellarioides* than to the conspecific Lawai population as shown in the PCoA plot and the network (Figure 5B).

### 3.4 Introgression Versus Incomplete Lineage Sorting to Explain Cytonuclear Discordance

Among the scenarios considered, introgression was strongly supported over incomplete lineage sorting as the reason for cytonuclear discordance, but we could not readily distinguish which of the two introgression models was more likely. When comparing all three models, the posterior probability was 0.551 in favor of the model of introgression into an ancestral WR population (scenario 3), but combining the two introgression models for comparison to the ILS model increased the posterior probability to 0.769 (and 362/500 votes) while still favoring introgression. The robustness of our data to support introgression is also supported by a low global out‐of‐bag (OOB) error rate of 0.086. The most important factors contributing to the selected model of introgression were linear discriminant analysis axis 1 (LDA1), followed by admixture mean loss (AML) values, suggesting that model choice was strongly driven by the signatures of shared alleles before and after hybridization. Point estimates of demographic parameters estimated from scenario 3 are reported in Table 6. These values suggest rather recent introgression into the WR populations (t = 1407 generations, 95% CI = 565–5024) and a moderate rate of gene flow (*r* = 0.36, 95% CI = 0.108–0.883) between *S. stellarioides* and the WR populations.

**TABLE 6:** Point estimates (median) and 95% CI values for demographic parameters estimated in ABC‐RF analyses for the scenario involving introgression from *Schiedea stellarioides* into an ancestral WR population of *Schiedea spergulina* .

Parameter	Median	95% CI
N Wai'alae	4260	1206–8000
N Kawaiiki	5234	464–9479
N Lawai	3077	755–8434
N WR1	4861	972–9150
N WR2	3735	447–9538
ra	0.363	0.108–0.883
t1	232	52–463
t2	1407	565–5024
t3	22,658	10,888–76,999

*Note: N* = ancestral population size, ra = rate of introgression, t1 = timing (generations) of coalescence of conspecific populations of *S. stellarioides* and coalescence of WR1 and WR2, t2 = timing of introgression between the *S. stellarioides* and the WR population, t3 = timing of coalescence of the two species.

## 4 Discussion

### 4.1 Lack of Nuclear Genetic Admixture Despite Chloroplast Introgression Between *Schiedea spergulina* and *S. stellarioides*

Despite the small number of loci considered in this study, there is a strong signal of introgression between *S. spergulina* and *S. stellarioides* that is present in the chloroplast genome. However, little evidence of nuclear admixture remains between these species. While Willyard et al. (2011) presented evidence of chloroplast introgression based on cytonuclear discordance between nuclear and chloroplast phylogenies for these taxa, they were unable to address the potential for nuclear introgression for these species. Results of multiple analyses on the nuclear microsatellite data, including the PCoA, network, (Figure 5A), AMOVA (Table 3), and STRUCTURE (Figure 4A), are consistent in supporting two groups that correspond with traditional species boundaries. AMOVA indicated as much as 57% of the variation was distributed between the two species. The chloroplast microsatellite data (Figure 5B), in contrast, support the idea of strong introgression of the plastid genome for the Waimea Canyon populations of *S. spergulina* , but not the Lawai population. This is not an unexpected result as chloroplast introgression with simultaneous lack of nuclear introgression has been well documented in many other plant species (Brunsfeld et al. 1992; Dorado et al. 1992; Riesberg et al. 1991; Riesberg and Soltis 1991; Smith and Sytsma 1990; Wendel et al. 1991). Our findings of no migration (Table 5), and thus no nuclear admixture, corroborate results from the genus‐wide phylogenetic analysis of three nuclear loci by Willyard et al. (2011), which provided strong support for placement of *S. stellarioides* in the sect. *Mononeura* (MII) clade and *S. spergulina* in the sect. *Schiedea* (S clade), and sister to all other species in the sect. *Schiedea* clade.

### 4.2 Extent and Direction of Chloroplast Introgression at the Population Level

Chloroplast microsatellites present a striking contrast to the results from the nuclear microsatellites, with PCoA and network results showing that all sampled individuals of the two Waimea Canyon populations of *S. spergulina* are more similar to chloroplast types observed in *S. stellarioides* (Figure 5B). ABC‐RF analyses suggest that introgression is much more likely to be the reason for the cytonuclear discordance than incomplete lineage sorting. Although we did not test convergent selection as another possible explanation for cytonuclear discordance, we suggest that it is unlikely that selection would target multiple non‐coding regions in a similar manner in two species that have discordant nuclear genomes as evidenced by our work and that of Willyard et al. (2011).

Random forest analysis estimated the rate of gene flow between *S. stellarioides* and the WR populations of *S. spergulina* as 0.363 (Table 6). Even at the lower estimated bound for this parameter (0.108), the results suggest moderate introgression between the two species. This likely explains why all WR1 and WR2 samples share similar chloroplast haplotypes. Willyard et al. (2011) concluded that, based on long branches in the plastid phylogeny for the *S. spergulina* clade, historical rather than recent introgression is more likely. However, the data presented here suggest a more recent time for introgression, approximately 1407 generations ago. Considering an average generation time of 5 years for *Schiedea* (Wallace et al. 2009), this would indicate that the species inter‐bred approximately 7000 years ago.

By comparison to the population of *S. spergulina* from Lawai, we are also able to conclude that the direction of introgression was likely from *S. stellarioides* into *S. spergulina* . The Lawai population may retain the ancestral chloroplast genome for *S. spergulina* . The potential for chloroplast introgression from *S. stellarioides* to the more inland, Waimea Canyon populations of *S. spergulina* is also supported by past distributions of both species which highly support the idea of past sympatry or at least parapatry. Both *S. spergulina* and *S. stellarioides* had much broader distributions in the past than they currently do as seen in Figure 1 and Figure 2. The range of the former species included most of the canyons of western Kauai, including the Olokele, Waimea and Waialae Canyons, Kalalau, Hipalau, Hanapepe, ridges west of Wahiawa on the central plateau of Kauai and the Lawai Valley. The populations of *S. spergulina* may even display morphological differentiation. Wagner et al. (1999) recognized two varieties of *S. spergulina* , based on presence of hairs in the populations from Olokele and Waimea Canyons, while populations from Hanapepe, Wahiawa and Lawai are glabrous and correspond to var. *leiopoda* (Wagner et al. 1999). *Schiedea stellarioides* occurred historically on the western side of Kauai from Waimea, Opaewela, Kaholuamanu and above Hanakapiai Beach along the Na Pali Coast, and with a single, disjunct historical collection from Haupu Ridge in southeastern Kauai. Historically overlapping ranges include localities in Waimea and Waialae and along the Na Pali coast ridges and valleys.

Massive asymmetrical introgression of plastid genomes has frequently been reported for species colonizing new habitats (Currat et al. 2008; Rieseberg et al. 2007). Simulations by Currat et al. (2008) suggest that introgression of neutral genes can occur during the invasion of an occupied territory if interbreeding is not inhibited between species. In their simulations, unidirectional plastid introgression always occurred from the resident species into the invading species. Conditions that promote unidirectional introgression may be especially common on islands with rapidly radiating clades and young and dynamic habitats. Indeed, cytonuclear discordance due to chloroplast introgression has been reported for several Hawaiian plant lineages (Baldwin et al. 2021; Dunbar‐Co et al. 2008; Knope et al. 2020; Yang et al. 2018). The similarity of plastid haplotypes of the Waimea Canyon populations of *S. spergulina* to those from *S. stellarioides* suggests that the former species expanded into the range of *S. stellarioides* and acquired its plastid genome.

Breeding systems are also likely to have an effect on the likelihood of chloroplast introgression (Tsitrone et al. 2003). *Schiedea stellarioides* is a hermaphrodite and is presumed to be pollinated by insects, while *S. spergulina* is fully dioecious and wind‐pollinated. If hybrid offspring of *S. stellarioides* in the upland Waimea area were pollinated by invading *S. spergulina* , then they should carry the chloroplast genome of *S. stellarioides* . Tsitrone et al. (2003) presented a model in which incompatibilities between chloroplast and the new nuclear genome in hybrids could lead to male‐sterile individuals that could serve as the female parent, with energy allocated entirely to female function resulting in increased seed production.

Other explanations for plastid introgression suggest that introgression may occur in species whose populations are declining if they are in close geographic proximity to a more common close relative, especially if mutational load is occurring in the rare species (Sloan et al. 2017). Our results suggest evidence of genetic bottlenecks for all populations of both species. There is, additionally, historical evidence that *S. stellarioides* has undergone a significant population bottleneck, as it had not been seen since 1916 and had been thought to be extinct until rediscovered in 1991. Populations of both species have experienced significant declines in the last century.

Alternative models are that positive selection on plastid haplotypes with higher fitness in different habitat conditions such as wetter vs. drier could provide an explanation for chloroplast introgression and fixation in certain populations. Sambatti et al. (2008) report differential survival of plastid haplotypes in *Helianthus* hybrids in xeric or mesic conditions supporting the idea that plastid haplotypes may provide some selective advantage in different environmental conditions. Likewise, Lee‐Yaw et al. (2019) further report evidence for positive selection on plastid genes, and that certain haplotypes tend to be associated with mesic or xeric habitats. Baldwin et al. (2021) describe a history of transitions from drier to wetter habitats in Hawaiian silverswords and suggest that the plastid phylogeny better documents potential back migrations into such habitats than what could be inferred from nuclear data alone. *Schiedea spergulina* is known to inhabit dry shrubland and cliffs, with the Waimea Canyon populations the most inland and mesic locations, whereas *S. stellarioides* inhabits more diverse mesic forest and more upland environments. The difference in habitat types between these two species is intriguing and suggests that plastid haplotypes of the more mesic *S. stellarioides* may have had some selective advantage for the invading populations of *S. spergulina* into a more inland and mesic environment, potentially leading to selection on the *S. stellarioides* chloroplast haplotype in the nearby Waimea Canyon populations of *S. spergulina* .

### 4.3 Genetic Diversity and Contrasting Genetic Structure of *Schiedea stellarioides* and *S. spergulina*

The nuclear genetic structure of these two species of *Schiedea*, connected only by a distant historical plastid introgression, contrasts strikingly, indicating that different forces are shaping the recent contemporary genetic structure of these species on Kauai. The nuclear genetic structure of populations of *S. stellarioides* , on one hand, based on STRUCTURE (Figure 4A,C) and PCoA (Figure 5), shows lack of population subdivision. These populations are located relatively close to one another (within five kilometers of each other; see map, Figure 1), along adjacent ridges near Waialae Falls on Kauai, so the potential for gene flow between them is high. In contrast, populations of *S. spergulina* show strong evidence of population subdivision at the nuclear loci and adhere more closely to expectations of allopatric divergence of populations. The results presented here do support the idea that the geographically isolated populations of *S. spergulina* are well differentiated, with overall species *F*_ST_ = 0.1419, and in particular, the Lawai population is significantly differentiated from other populations with an *F*_ST_ = 0.459 (Table 2) and appears relatively distinct based on the results of STRUCTURE analyses (Figure 4), PCoA, and the network analysis (Figure 5).

Differences in population genetic structuring between these two species of *Schiedea* may be influenced by breeding system and historical factors. Given the hermaphrodite breeding system of *S. stellarioides* , we would expect stronger genetic structuring between populations, due to the possibility of higher selfing rates than expected in a completely outcrossing species, despite close proximity of its populations. However, the effects of inbreeding could counteract those effects. *Schiedea stellarioides* has somewhat lower observed heterozygosity (0.234 and 0.241 in the Waialae and Kawaiki populations, respectively, Table 2), and moderate levels of inbreeding (0.287 for the Waialae population, 0.289 for the Kawaiki population, Table 2). A population bottleneck would be consistent with *S. stellarioides* extirpation in recent times from historical localities, and more recent rediscovery of two small populations in the vicinity of Waialae Ridge in the interior plateau of Kauai. The reduced observed heterozygosity, together with the moderate *F*_IS_ values reported here, supports the idea that moderate levels of inbreeding have occurred for populations of this species and might mitigate the effects of breeding system on population genetic structure. Inbreeding in small, fragmented populations of this species could be a force that mediates population genetic structure based on breeding system. Strong differentiation of populations based on nuclear microsatellites in *S. spergulina* is also counter to expectations based on the dioecious breeding system for this species. Dioecious species are obligate outcrossers that would maintain population connectivity. However, the strong genetic structuring results are likely to be highly skewed given the reduction in number of populations from historic times, population bottlenecks and current geographic distribution of the remaining populations.

### 4.4 Implications for the Conservation of *Schiedea spergulina* and *S. stellarioides*

Currently, 15 of the 36 extant species in the genus *Schiedea* are listed as endangered or threatened by the United States Fish and Wildlife Service. The main threats to these species are habitat destruction caused by humans and other feral animals such as pigs and goats. Many populations of *S. spergulina* that were easily found even 25 years ago are thought to have been extirpated from these remote areas due to feral pigs and goats. Goats are now a major threat to both species, as goats can easily scale the cliffs and hillsides that the last remaining populations of these species now occupy.

Conservation of these two species is both important and difficult, due to their small population sizes and overall small total numbers for the species. This research indicates that populations of *S. stellarioides* harbor low levels of genetic diversity. Low levels of genetic diversity are also apparent in *S. spergulina*; however, individual populations are well differentiated from one another. In particular, the population of *S. spergulina* in the Lawai Valley, which aligns with the formerly recognized var. *leiopoda*, may deserve a separate designation because of its genetic uniqueness from conspecific populations in the Waimea Canyon. Fortunately, this particular population is among the most protected from human destruction as it is contained within the property of the National Tropical Botanical Gardens. Because other populations of the species recognized as var. *leiopoda* could not be examined in this study due to their local extirpation in all other parts of its range, we are limited in the statements we can make regarding the genetic as well as taxonomic uniqueness of Sherff's variety *leiopoda*. Further work on the distinction and possible re‐designation of the Lawai Valley population as var. *leiopoda* should be addressed in future research. Regardless, the paucity of extant populations of both species, combined with the genetic results presented in this study, indicate a critical need to preserve the genetic integrity of all populations for the continued survival of both species.

## Author Contributions

**Molly Nepokroeff:** conceptualization (equal), funding acquisition (lead), methodology (equal), project administration (lead), resources (lead), supervision (lead), writing – original draft (supporting), writing – review and editing (lead). **Bridget Jacobs:** conceptualization (equal), formal analysis (equal), investigation (equal), methodology (equal), visualization (equal), writing – original draft (lead). **Lisa Wallace:** conceptualization (equal), data curation (lead), formal analysis (equal), funding acquisition (supporting), investigation (equal), methodology (equal), project administration (supporting), resources (supporting), supervision (supporting), visualization (equal), writing – review and editing (equal).

## Funding

This work was supported by the National Science Foundation, DEB‐0445410 and J. Robert Stiffler Endowment at Old Dominion University for funding.

## Conflicts of Interest

The authors declare no conflicts of interest.

## Data Availability

Genetic data: Individual genotype data for nuclear and chloroplast microsatellites are available on Dryad at the following link: https://doi.org/10.5061/dryad.4qrfj6qsn.
